# Protective effect of quercetin on skeletal and neural tube teratogenicity induced by cyclophosphamide in rat fetuses

**Published:** 2016-06-15

**Authors:** Mahmood Khaksary Mahabady, Mohammad Reza Gholami, Hossein Najafzadeh Varzi, Abolfazl Zendedel, Mona Doostizadeh

**Affiliations:** 1 *Department of Anatomy and Embryology, Faculty of Veterinary Medicine, Shahid Chamran University of Ahvaz, Ahvaz, Iran; *; 2 *Razi Herbal Medicines Research Center and Department of Anatomy, Lorestan University of Medical Sciences, Khorramabad, Iran; *; 3 *Department of Pharmacology, Faculty of Veterinary Medicine, Shahid Chamran University of Ahvaz, Ahvaz, Iran; *; 4 *Department of Internal Medicine, Lorestan University of Medical Sciences, Khorramabad, Iran.*

**Keywords:** Cyclophosphamide, Fetus, Quercetin, Rat, Teratogenicity

## Abstract

Cyclophosphamide (CP) is a drug commonly used to treat neoplastic disease and some autoimmune diseases. It is also a well-known and well-studied teratogen causing a variety of birth defects in fetuses of pregnant women treated with the drug. There are many reports that show the adverse effects of CP can be decreased by use of antioxidant drugs. It appears that, quercetin has antioxidant effect. The aim of this study was prevention or decrease of teratogenicity of CP in fetuses of rats by quercetin. This study was performed on 35 pregnant rats divided into six groups. Control group was received normal saline (5 mL kg^-1^, intraperitoneally) and 2-6 groups received a single dose of CP (15 mg kg^-1^), a single dose of quercetin (75 or 200 mg kg^-1^), CP plus quercetin (75 or 200 mg kg^-1^) intraperitoneally at 9^th^ day of gestation, respectively. Fetuses were collected at 20^th^ day of gestation and after determination of weight and crown rump length were stained by alizarin red – alcian blue method and skeletal system were examined by stereomicroscope. The results showed that the cleft palate, exencephaly, spina bifida and omphalocele incidence were 55.56%, 27.77%, 33.34% and 11.11%, in fetuses of rat that received only CP, respectively. However, it decreased to 16.00%, 16.00%, 16.00% and 8.00% by quercetin (75 mg kg^-1^) and so to 12.90%, 12.90%, 6.45% and 3.28% by quercetin (200 mg kg^-1^), respectively. On the basis of results, quercetin significantly can decrease teratogenicity induced by CP.

## Introduction

Some chemical agents and drugs can induce terato-genic effects and abortion.^1^ Developmental defects are major health problems as in the USA 3.00 to 5.00% of fetuses have congenital abnormality.^2^ It is estimated that 7.00 to 10.00% of human anatomic anomalies result from the disruptive actions of drugs, viruses, and other environmental factors.^3^ De Sanntis *et al.* also estimated that defects attributable to drug therapy represent about 1.00% of congenital defects of known etiology.^4^

Cyclophosphamide (CP) is a drug commonly used to treat neoplastic disease and some autoimmune diseases. It is also a well-known and well-studied teratogen causing a variety of birth defects in the fetuses of pregnant women treated with the drug.^5^

Cyclophosphamide, a nitrogen mustard compound, is a member of the group of cytostatic alkylating agents and has several toxic effects including hemorrhagic cystitis.^6^ Metabolites of CP, especially acrolein modulates its toxic effects.^3^ In order to cause teratogenesis, CP must be bio-activated through a process involving oxidase enzymes that convert it into its active metabolites, phosphoramide mustard and acrolein.^7^ Phosphoramide mustard acts to inhibit DNA synthesis and causes cross-links in the existing DNA resulting in cell death, and acrolein is thought to be responsible for some of the side effects of CP chemotherapy, such as cystitis.^3^ Although the mechanism of teratogenesis is still debated, it is believed that generation of reactive oxygen species (ROS) through these metabolites plays a role in CP-induced malformations.^7^^,^^8^

Previous studies in rodents have shown that exposure to CP during organogenesis caused an embryonic and fetal resorption, growth retardation, or multiple anomalies, including exencephaly and limb and skeletal defects.^9^ Free radicals or ROS are by-products of the breakdown of many drugs.^10^ The exposure of the embryo or fetus to ROS is normally carefully timed so that exposure occurs when antioxidant levels are also high, potentially decreasing the duration of the ROS signal and enabling the cell to repair damage to its DNA.^10^ However, exposure to excessive levels of ROS without sufficient antioxidant presence can cause brain and spinal cord defects, embryonic death, or skeletal malformations.^10^

Oxidative stress can be prevented by antioxidants known to be effective *in vitro* for protection against conditions associated with oxidative damage through radical scavenging.^11^ Antioxidant agents such as squalene,^12^ melatonin,^13^ glutamine,^14^ and S-allylcysteine^15^ have protective actions against CP-induced toxicity. Thus, a combination of the drug delivered together with a potent antioxidant may be appropriate to reduce the toxic side effects of CP.

On the other hands, quercetin, commonly named sophretin and meletin, is a herbal flavonoid found in abundance in apple, onion, tea, green tea leaf, straw-berries, broccoli and other plants. Quercetin also has anti-inflammatory, anti-bacterial and antioxidant and is used in the prevention of cancer and cardiovascular disease.^16^ Quercetin is a powerful antioxidant and free radical scavenger, more powerful than other antioxidants such as vitamin E, vitamin C, which prevents lipid peroxidation.^17^ Quercetin supplementation to the diet of pregnant mice reduces fetal malformations caused by methylnitrosourea such as fingers and toes abnormalities. It induces fetal abnormalities via oxidative stress and free radicals.^18^

Cyclophosphamide can be teratogenic via oxidative stress. So far, the effects of quercetin have not been studied on CP-induced skeletal malformations in rat fetuses. In the present study, the prophylactic effect of quercetin on CP –induced neural tube defects and skeletal malformations in rat fetuses was evaluated.

## Materials and Methods

Male and female healthy Wistar rats, 3 to 4 months of age, weighting 200 to 220 g were purchased (Jundi-shapour Laboratory Animal Center, Ahvaz, Iran) and housed individually (males) or in 10 per poly-carbonate cage (females) for a 2-week acclimation period. Rats were fed *ad libitum* by standard laboratory pellet (Pars Khurak-e-Dam, Tehran, Iran) and tap water. A 12 hr light: 12 hr dark was exercised. Room temperature was at 23 ± 2 ˚C with a relative humidity of 45.00 to 55.00%. This experimental study was conducted in Department of Basic Sciences of Faculty of Veterinary Medicine of Shahid Chamran University (Ahvaz, Iran). The animal care was provided under the supervision of a qualified veterinarian. 

Females were mated overnight with males. Pregnancy was ascertained the next morning by presence of a vaginal plug, and this time was designated as gestational day (GD) 0. Ten rats were used in each group (total 60 rats) but 35 rats were harvested as pregnant. Thus, animals in each group were not equal. Pregnant rats (n = 35) were randomly divided into six groups (28 pregnant rats in treatment groups, seven pregnant rats in control group) and treated as follows:

Group 1 (control group): Normal saline (5 mL kg^-1^) was administrated to pregnant rats for inducing similar condition (injection and handling) to other groups.

Group 2 (CP group): A single dose of CP (15 mg kg^-1^) was administrated intraperitoneally (ip) at 9^th^ day of gestation.^19^

Group 3 (quercetin 75): A single dose of quercetin (75 mg kg^-1^, ip) was administrated at 9^th^ day of gestation.^20^

Group 4 (quercetin 200): A single dose of quercetin (200 mg kg^-1^, ip) was administrated at 9^th^ day of gestation.^20^

Group 5 (CP + quercetin 75): CP (15 mg kg^-1^, ip) plus quercetin (75 mg kg^-1^, ip) was administrated at 9^th^ day of gestation.

Group 6 (CP + quercetin 200): CP (15 mg kg^-1^, ip) plus quercetin (200 mg kg^-1^, ip) was administrated at 9^th^ day of gestation.

Cyclophosphamide (Baxter Oncology GmbH, Halle, Germany) and quercetin (Sigma-Aldrich, St. Louis, USA) were purchased. The animals were euthanized by diethyl ether and cervical dislocation at 20^th^ day of gestation. Following laparotomy, the uterus was exteriorized and the number and location of fetuses and resorption were noted, then their weight and crown - rump length (CRL) were measured. Individual fetuses were examined carefully for external anomalies then were stained in a mixture of 0.14% alcian blue and 0.12% alizarin red S in ethanol and glacial acetic acid. Fetuses were then macerated in 2.00% KOH, cleared and hardened in 1:1 glycerin and distilled water, and stored in pure glycerin^21^ and investigated by stereo-microscope (Model SMZ200; Nikon, Tokyo, Japan) for skeletal malformations. The incidence of skeletal mal-formations was determined and compared between groups.

Statistical significance between groups was determined using SPSS (Version 16; SPSS Inc., Chicago, USA) and compared by one way analysis of variance (ANOVA) followed by least significant difference (LSD) post hoc comparison. The minimum level of significance was *p *< 0.05.

## Results

No maternal deaths were observed throughout the course of this study. Likewise, the dose of CP used in this investigation was well tolerated by the dams.

**Fig. 1:**
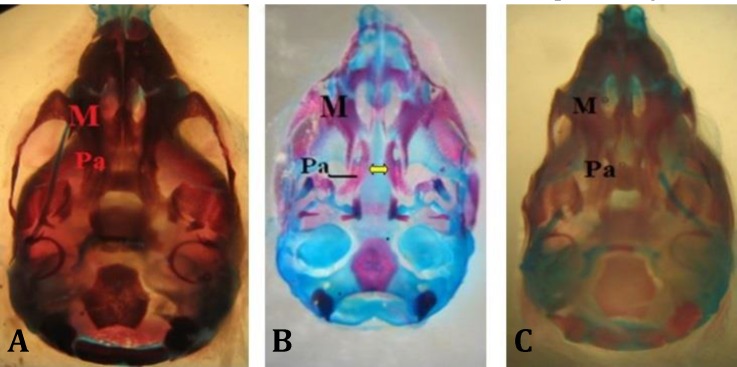
Ventral view of skull of rat fetuses of GD 20, stained with alizarin red S-alcian blue. **A)** Normal palatine bone (control); **B)** Cleft palate induced by CP (CP) (arrow); C**)** Normal palatine bone in group that received CP along with quercetin. M: Maxilla; Pa: Palatine

**Fig. 2:**
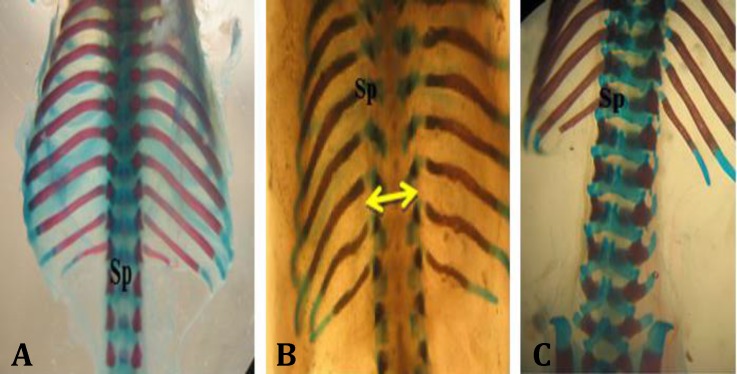
Dorsal view of vertebral column of gestation at 20^th^ day fetal rat, stained with alizarin red- alcian blue. **A)** Normal vertebral column (control); **B)** Spina bifida (arrow) induced by cyclophosphamide (CP) ; **C)** Normal vertebral column in group that received CP along with quercetin. SP: Spinous process

**Fig. 3:**
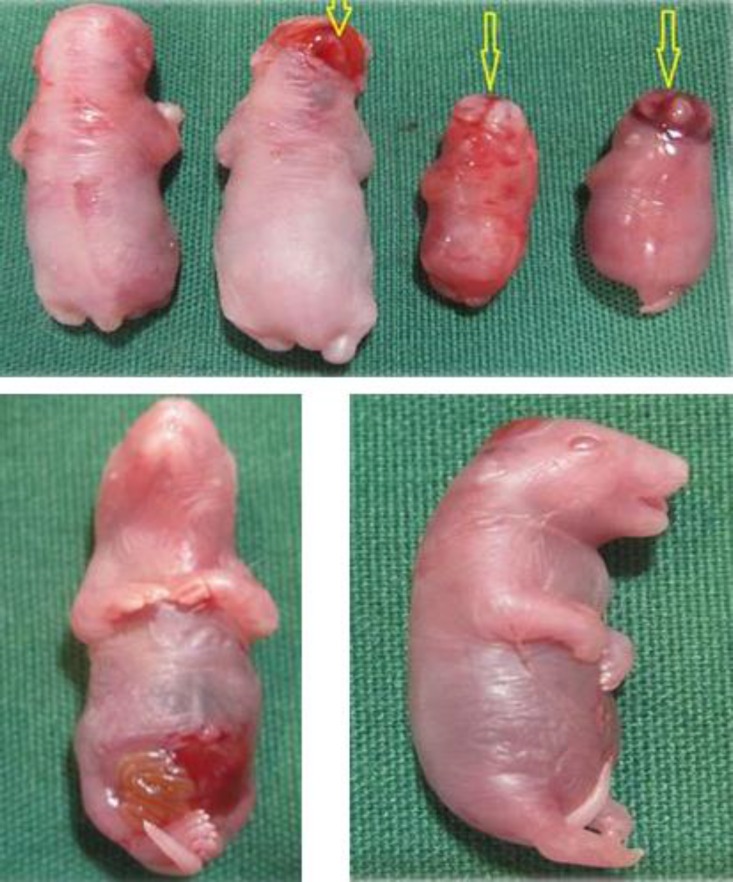
Some anomalies in fetuses of rats. Normal fetus (up-left), arrows indicate exencephaly (up-right), omphalocele (left-down) and open eye (right-down

Forty-seven fetuses were obtained from seven rats of control group. No macroscopic anomalies were observed in the control animals. In the control group palatal closures of fetuses were normal at gestational day 20 (i.e., palatal shelves had grown vertically on the sides of the tongue, then horizontally to meet and fuse (Fig. 1A). Cyclophosphamide induced cleft palate (Fig. 1B), spina bifida (Fig. 2), exencephaly (Fig. 3) and omphalocele (Fig. 3) at 55.56%, 27.77%, 33.34% and 11.11% incidences, respectively. 

However, it was decreased to 16.00, 16.00, 16.00 and 8.00% in group which received CP plus quercetin (75 mg kg^-1^) and so to 12.90, 12.90, 6.45 and 3.28%, in the group which received CP plus quercetin (200 mg kg^-1^), respectively. No maternal death or abortion occurred in any experimental groups. There were not any aborted fetuses in any groups but percentage of resorbed fetuses were 4.09, 56.09, 7.89, 7.14, 30.56 and 20.52% in groups that received normal saline, CP (15 mg kg^-1^), quercetin (75 mg kg^-1^), quercetin (200 mg kg^-1^), CP plus quercetin (75 mg kg^-1^), and CP plus quercetin (200 mg kg^-1^), respectively; therefore, quercetin decreased resorption rate.

**Fig. 4:**
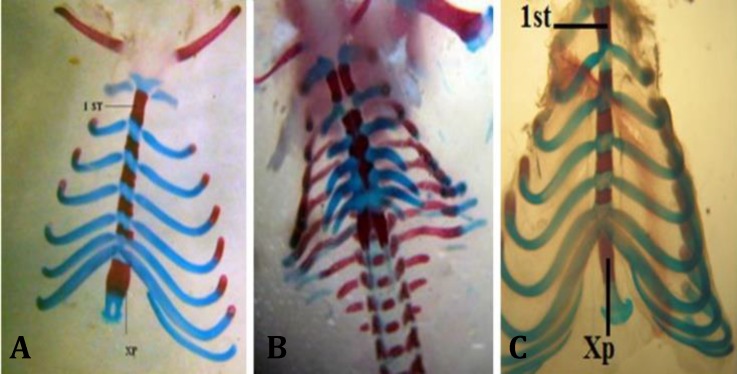
Dorsal view of sternum of gestation at 20^th^ day fetal rat, stained with alizarin red - alcian blue. **A)** Normal sternum (control); **B)** fused sternebrae induced by cyclophosphamide (CP); **C)** Normal sternum in group that received CP along with quercetin. 1st: First sternebrum; Xp: Xiphoid process

**Fig. 5:**
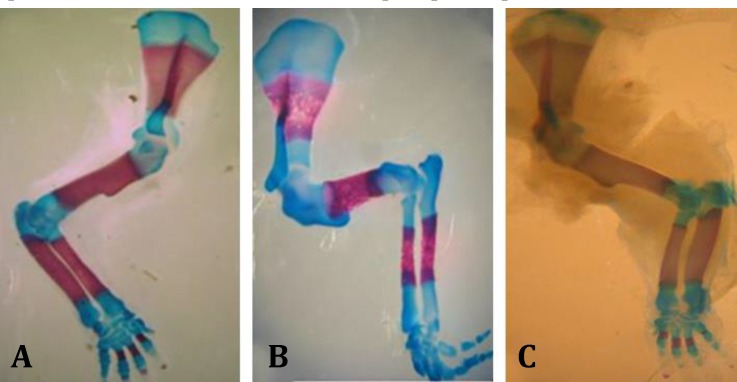
Lateral view of limbs of gestation 20^th^ day fetal rat, stained with alizarin red - alcian blue. **A)** Normal forelimb; **B)** Delay ossification in forelimb; **C)** Normal forelimb in in group that received cyclophosphamide along with quercetin

Open eye and omphalocele, delay ossification in forelimb and several anomalies in sternum were observed (Figs. 3, 4 and 5). Teratogenicity in groups that received CP was similar to groups that received CP plus quercetin, but incidence was lower (Table 1). These anomalies were not observed in animals treated with quercetin. Mean weight and CRL (*p* < 0.001) were significantly decreased in the group which received only CP. The means weight and length in groups that received CP plus quercetin was greater than the group received only CP except with CP plus quercetin (200 mg kg^-1^), (Table 2). The mean weight and CRL in the group that received quercetin were significantly decreased in comparison with control group (*p* < 0.001).

**Table 1.** Incidence (%) of anomalies in fetuses of groups

**Anomaly**	**Group 2**	**Group 5**	**Group 6**
**Cleft palate**	55.56	16.00	12.90
**Exencephaly**	27.77	16.00	12.90
**Spina bifida**	33.34	16.00	6.45
**Open eye**	27.77	0.00	0.00
**Omphalocele**	27.77	8.00	3.22
**Delayed ossification in forelimb**	33.34	16.00	3.22
**Fused sternebrae**	55.56	12.00	9.67

**Table 2:** Mean weight and crown rump length in rat fetuses of groups. Data are presented as mean ± SEM

**Groups**	**Litters (No.)**	**Implantations** **(No.)**	**Resorbed fetuses (%)**	**Live fetuses (%)**	**Fetal length (mm)**	**Fetal weight (g)**
**Control**	7	49	2(4.09)^a^	47(95.91)^a^	37.3 ± 0.31^a^	4.73 ± 0.07^a^
**Cyclophosphamide (CP; 15 mg kg** ^-1^ **)**	6	41	23(56.09)^b^	18(43.90)^c^	27.83 ± 1.34 c	1.96 ± 0.20^c^
**Quercetin (mg kg** ^-1^ **)**	6	38	3(7.89)^a^	35(92.11)^a^	34.64 ± 0.68^b^	3.12 ± 0.10^b^
**Quercetin (200 mg kg** ^-1^ **)**	6	42	3(7.14)^a^	39(92.86)^a^	29.13 ± 0.69^b^	3.29 ± 0.08^c^
**CP + quercetin (75 mg kg** ^-1^ **)**	5	36	11(30.56)^c^	25(69.44)^b^	32.94 ± 0.53^b^	2.46 ± 0.13^e^
**CP + quercetin (200 mg kg** ^-1^ **)**	5	39	8(20.52)^c^	31(79.48)^b^	33.85 ± 0.45^c^	2.24 ± 0.10^ce^

abc Different letters indicate significant differences each parameter between groups (*p *≤ 0.05).

## Discussion

Since there are not data available on quercetin on the teratogenicity of CP in rat fetuses. In the present study, for first time, the effect of quercetin on teratogenicity of CP in rat fetuses was evaluated. We demonstrated CP, at dose of 15 mg kg^-1^, decreased weight and length and produced cleft palate (55.56%), exencephaly (27.77%), spina bifida (33.34%) and omphalocele (11.11%) among all fetuses. The results presented here show that quercetin administration during the gestational period has a partial protective effect on CP-induced terato-genesis (decreasing the frequencies of exencephaly, cleft palate, spina bifida and omphalocele). In the present study, quercetin reduced the frequency of incidence of neural tube and skeletal fetal defects. Quercetin with dose of 200 mg kg^-1^ was more effective on decreasing the incidence of neural tube and skeletal fetal defects than 75 mg kg^-1^, but it is not significant. 

It is well known that CP causes fetal defects in diverse species of animals including mice, rats, hamsters, and rabbits as well as humans.^22^ In the present study, a single intraperitoneal administration of CP (15 mg kg^-1^) on GD9 caused significant growth retardation and morpho-logical alterations in rat fetuses.

Gibson and Becker reported CP-induced terato-genicity in mice. They used intraperitoneal CP at dose 5 to 20 mg kg^-1^ in mice in one of 9^th^ to 14^th^ day of gestation. They observed the CP could produce teratogenicity in 67.30% of fetuses with 20 mg kg^-1^.^23^ They determined fetal defects similar with our study including cleft palate, exencephaly. These anomalies were decreased by 75 mg kg^-1^ and 200 mg kg^-1^ quercetin, respectively. They also determined fetal weights and crown rump lengths similar with our study reduced significantly by CP. In present study fetal weights and crown rump lengths were increased by 75 mg kg^-1 ^and 200 mg kg^-1^quercetin, respectively in comparison with CP.

Sloth and Hales evaluated effect of mesna on CP-induced teratogenicity. They used CP at dose 10 and 15 mg kg^-1^ in rats in 13^th^ day of gestation. They observed the CP could produce teratogenicity in 50.00% and 100% of fetuses with 10 and 15 mg kg^-1^, respectively.^19^ They determined fetal defects similar with our study including cleft palate, exencephaly, open eye and limb defects. These anomalies were decreased by 75 mg kg^-1^ and 200 mg kg^-1^ quercetin, respectively.

Logsdon *et al*., reported CP at dose 20 mg kg^-1 ^in mice in on 10^th^ day of gestation could produce teratogenicity and exposure of a developing mammal to moderate doses of green tea as antioxidant can modulate the effects of exposure to CP during development, possibly by affecting biotransformation, while a higher GTE dose tended to exacerbate the developmental toxicity of CP.^24^ They determined fetal defects similar with our study including fused or dumbbell-shaped vertebral centra and limb defects. These anomalies were decreased by 75 mg kg^-1^ and 200 mg kg^-1 ^quercetin, respectively. They also determined fetal weights similar with our study reduced significantly by CP. In the present study, fetal weights increased using 75 mg kg^-1^ and 200 mg kg^-1 ^quercetin, respectively.

Najafzadeh Varzi and Khaksari Mahabadi evaluated effect of mesna and *Echinacea* *purpurea* on CP-induced teratogenicity. They used intraperitoneal CP at dose 15 mg kg^-1^ in rats on 13^th^ day of gestation.^25^ They determined fetal defects similar with our study including cleft palate, exencephaly, open eye and limb defects. These anomalies decreased by 75 mg kg^-1 ^and 200 mg kg^-1^ quercetin, respectively.

Oxidative stress in any tissue results from an imbalance between the production of ROS such as super-oxide anion, hydrogen peroxide, and the hydroxyl ion. A number of teratogens including anti-neoplastic agents have been shown to initiate potentially embryopathic oxidative stress.^26^ Cyclophosphamide exposure increases ROS production, suggesting that biochemical and physio-logical disturbances may result from oxidative stress.^27^

Quercetin decreased CP teratogenicity in our study. However, this property of quercetin was reported in other related studies. Quercetin (75 mg kg^-1^) had beneficial effect on serum lipid and glucose profile and minimized the monosodium glutamate related toxic effects, which was associated to its antioxidant properties.^28^ Also, it has protected spinal cord against mechanism of inhibiting the activation of p38MAPK/iNOS signaling pathway and thus regulating secondary oxidative stress.^29^

Quercetin with the dose of 66 mg kg^-1 ^(low dose) and 333 mg kg^-1 ^(high dose) throughout gestation, decreased placental oxidative stress and fetal skeletal malformation induced by methylnitrosourea.^30^ Quercetin prevented renal tubular damage oxidative stress induced by chronic cadmium administration.^31^

Hydroxyurea caused abnormal development of mouse embryos which is also reduced by quercetin.^32^ Liang *et al.* reported that saturated fatty and lipid peroxidation related to fetal skeletal anomalies and quercetin (66 mg kg^-1 ^supplemented diet) significantly improved their defects probably by its antioxidant effect on placenta.^33^ This protective property of quercetin was demonstrated on all- trans-retinoic acid-induced teratogenicity when used at doses of 75 mg kg^-1 ^and 200 mg kg^-1 ^in rats on 8^th^ to10^th^ days of gestation.^20^

In conclusion, the results of these studies are in consistent with the results of a recent study showing the ability of quercetin to reduce the damage caused by oxidative agents. Results of our study showed the effects of quercetin on elimination of CP induced teratogenicity for the first time. Taken together, 15 mg kg^-1 ^CP on the 9^th^ day of pregnancy causes fetal malformations including cleft palate, exencephaly, spina bifida and skeletal abnormality in rat. Quercetin not only reduces skeletal abnormality but also protects weight and length abnormality of the fetus induced by CP. On the other hand, quercetin (200 mg kg^-1^) is more effective than quercetin (75 mg kg^-1^) in decreasing incidence CP-induced neural tube and skeletal defects in fetuses of rats. Therefore, antioxidant property of quercetin can protect the fetus against damage caused by CP.
